# Case of Fracture of the Skull

**Published:** 1859-10

**Authors:** H. D. La Cossitt

**Affiliations:** West Greenville, Mercer Co., Pa.


*Case of Fracture of the Skull*—On the 20th of March, 1858, James Mathers, age 36, citizen of this village, returning home from a fishing excursion, while assisting some others to draw a skiff into a canal-lock with ropes, standing on a board across the lock, his rope gave way, and he plunged head first, a distance of ten or fifteen feet, to the bottom of the lock; his head striking the miter sill, fracturing the left lateral and superior part of the cranium. He was insensible for a few moments. He was taken up on the tow-path, and soon came to his mind. He was not aware of the extent of the injury he received; and walked home a distance over a mile. When I arrived, he conversed as usual, and stated that his head felt strangely. The side of his face and neck were sore and stiff; and upon examination I found, over the left parietal bone, a puffy tumor of the integuments. I made a free opening with the scalpel, and the blood discharged. I passed in my finger, and found concussion with compression, requiring attention; the fractured part depressed was about the size of a Spanish dollar. I removed the hair, and dissected back the scalp, so as to lay bare the fracture. Then, with Hey's saw, I took out a piece of the sound part of the skull adjoining the fracture, in the form of the letter V, which enabled me, with the use of the lever and bone-plyers, to remove the fragments of the outer table; those of the inner table were of larger size, which I extracted by pieces. There was considerable blood discharged during the operation. After cleansing the wound, the scalp was brought together with sutures and adhesive straps. The union took place by the first intention. He was conscious at the time, and has since enjoyed his intellectual faculties. In this case there wâs no violence of reaction, and I used no antiphlogistic remedies. After the third day, I allowed him a reasonable diet. He recovered, and was on the street attending to his business twenty days after the accident. In former times, when there was a fracture of the cranium requiring the use of the trephine, it was thought that a rigid course of bleeding and depletion was necessary. I trust all these errors are abandoned, and medicines not administered unless the case demands.

I have performed the operation eleven times for fracture of the skull. Nine have recovered. It is important that every irritating substance be removed, and the operation performed as soon as circumstances will permit, when it is necessary. I must avail myself of this opportunity to acknowledge the able assistance rendered me during the operation in the above case by Dr. F. Donaldson, an intelligent physician of this village.

H. D. La Cossitt, M. D.

West Greenville, Mercer Co., Pa.,
*Feb.* 10, 1859.

